# *SLC41A1* is essential for magnesium homeostasis in vivo

**DOI:** 10.1007/s00424-018-2234-9

**Published:** 2018-11-12

**Authors:** Francisco J. Arjona, Femke Latta, Sami G. Mohammed, Michael Thomassen, Erwin van Wijk, René J. M. Bindels, Joost G. J. Hoenderop, Jeroen H. F. de Baaij

**Affiliations:** 1 Department of Physiology, Radboud Institute for Molecular Life Sciences, Radboud University Medical Center, Nijmegen, The Netherlands; 2 Department of Otorhinolaryngology, Donders Institute for Brain, Cognition and Behaviour, Radboud University Medical Center, Nijmegen, The Netherlands

**Keywords:** SLC41A1, Kidney, Magnesium, Membrane transport, Hypomagnesemia

## Abstract

Solute carrier family 41 member A1 (SLC41A1) has been suggested to mediate magnesium (Mg^2+^) transport by several in vitro studies. However, the physiological function of SLC41A1 remains to be elucidated. In this study, cellular Mg^2+^ transport assays combined with zebrafish *slc41a1* knockdown experiments were performed to disclose SLC41A1 function and its physiological relevance. The gene *slc41a1* is ubiquitously expressed in zebrafish tissues and is regulated by water and dietary Mg^2+^ availability. Knockdown of *slc41a1* in zebrafish larvae grown in a Mg^2+^-free medium resulted in a unique phenotype characterized by a decrease in zebrafish Mg content. This decrease shows that SLC41A1 is required to maintain Mg^2+^ balance and its dysfunction results in renal Mg^2+^ wasting in zebrafish larvae. Importantly, the Mg content of the larvae is rescued when mouse SLC41A1 is expressed in *slc41a1*-knockdown zebrafish. Conversely, expression of mammalian SLC41A1-p.Asp262Ala, harboring a mutation in the ion-conducting SLC41A1 pore, did not reverse the renal Mg^2+^ wasting. ^25^Mg^2+^ transport assays in human embryonic kidney 293 (HEK293) cells overexpressing SLC41A1 demonstrated that SLC41A1 mediates cellular Mg^2+^ extrusion independently of sodium (Na^+^). In contrast, SLC41A1-p.Asp262Ala expressing HEK293 cells displayed similar Mg^2+^ extrusion activities than control (mock) cells. In polarized Madin-Darby canine kidney cells, SLC41A1 localized to the basolateral cell membrane. Our results demonstrate that SLC41A1 facilitates renal Mg^2+^ reabsorption in the zebrafish model. Furthermore, our data suggest that SLC41A1 mediates both Mg^2+^ uptake and extrusion.

**Electronic supplementary material:**

The online version of this article (10.1007/s00424-018-2234-9) contains supplementary material, which is available to authorized users.

## Introduction

Magnesium (Mg^2+^) reabsorption in the kidney is key for Mg^2+^ homeostasis. Of the 2400 mg of Mg^2+^ filtered in the kidney on a daily basis in humans, 95–99% is recovered through tubular Mg^2+^ reabsorption [9]. Indeed, impaired renal Mg^2+^ handling results in a heterogeneous group of disorders that are characterized by hypomagnesemia (serum Mg^2+^ < 0.7 mmol/L) [9]. Patients suffer from fatigue, seizures, and muscle cramps. Over the last decade, studying hereditary forms of hypomagnesemia and urinary Mg^2+^ wasting has resulted in the elucidation of the molecular mechanisms that regulate renal Mg^2+^ reabsorption [9].

Mg^2+^ is filtered in the glomerulus and subsequently passively reabsorbed in the proximal tubule (PT, 25%) and thick ascending limb of Henle’s loop (TAL, 70%). The remainder is reabsorbed in the distal convoluted tubule (DCT), where Mg^2+^ reabsorption takes place in a transcellular manner via transient receptor potential melastatin type 6 (TRPM6) Mg^2+^ channels [27]. Mutations in *TRPM6* cause hypomagnesemia and secondary hypocalcemia (HSH) [25, 29]. Its membrane expression and channel activity are dependent on the epidermal growth factor (EGF), insulin, estrogen, flavaglines, and ATP [3, 5, 8, 23, 26]. TRPM6 is the apical gatekeeper for the regulation of renal Mg^2+^ reabsorption. However, the basolateral Mg^2+^ extrusion mechanism remains subject of debate, and no proteins have been characterized facilitating this process [9].

Recently, the solute carrier family 41 member A1 (SLC41A1) was suggested to facilitate cellular Mg^2+^ extrusion by operating as a Na^+^-Mg^2+^ exchanger [17, 20]. SLC41A1 is a distant homolog of the bacterial MgtE Mg^2+^ transporter and is widely expressed in the human body [24, 28]. In the kidney, SLC41A1 proteins are localized in the macula densa, TAL, and DCT [17], and target the basolateral membrane when expressed in a polarized renal epithelial cell line [17]. In addition, dietary Mg^2+^ was shown to regulate renal *Slc41a1* gene expression in mice [13]. Despite these evidences linking SLC41A1 with cellular Mg^2+^ transport, SLC41A1 has not been linked with the regulation of the Mg^2+^ balance in vivo, remaining unclear whether SLC41A1 is relevant for renal Mg^2+^ reabsorption.

In this study, we aimed to elucidate the physiological role of SLC41A1 by knockdown experiments in the zebrafish model. Moreover, the molecular function of SLC41A1 was studied by cellular Mg^2+^ transport experiments.

## Materials and methods

### Cloning

The full-length mouse *Slc41a1* transcript was amplified from a cDNA clone with GenBank accession number NM_173865 (Source BioScience, Nottingham, UK) using Phusion polymerase. The primer sequences contained *Asc*I and *Xba*I and are provided in Table 1. The amplicons were ligated into the pCINeo-IRES-GFP expression vectors containing an in-frame N-terminal HA-tag using the *Asc*I and *Xba*I restriction sites. The p.Asp262Ala mutation in mouse SLC41A1 (equivalent to the p.Asp263Ala mutation in the human SLC41A1 sequence) was introduced using the QuikChange site-directed mutagenesis kit (Agilent, Amstelveen, The Netherlands). For experiments in the zebrafish model, the SLC41A1 constructs were subcloned into pT7Ts vectors using the *Asc*I and *Xho*I restriction enzymes. All constructs were verified by sequence analysis. Throughout the manuscript, we followed the guidelines from HUGO, MGI, and ZFIN on gene nomenclature for human, mouse, and zebrafish genes, respectively.

**Table 1:** Primer sequences for cloning

Construct	Sequence	
pCINeo	5′-GGCGCGCCGATGTCCTCTAAGCCAG-3′	FW
pCINeo	5′-GCTCTAGAGCCTAGTCCCCAACATC-3′	RV
p.Ile98Phe	5′-GAGACCTCCTTTTCCTTCGGGCTGCAAGTACTG-3′	FW
p.Ile98Phe	5′-CAGTACTTGCAGCCCGAAGGAAAAGGAGGTCTC-3′	RV

*FW* forward primer, *RV* reverse primer

### Tissue distribution of *slc41a1* gene expression and Mg^2+^ challenges in zebrafish adults and larvae

Zebrafish from the Tupfel long-fin (TLF) strain were used for experimentation. For the study of the tissue distribution of *slc41a1* in adult zebrafish tissues, three females and three males were dissected following anesthesia (0.1% (*v*/*v*) 2-phenoxyethanol (Sigma Chemical Co., St. Louis, USA)). Brain, ovary, gills, testis, heart, spleen, kidney, gut, operculum, scales, and liver tissues were collected and stored at − 80 °C until analysis. The expression of *slc41a1* in the pronephric tissue was determined in 120 hours post-fertilization (hpf) zebrafish which were grown in E3 medium (0.33 mmol/L MgSO_4_ 5 mmol/L NaCl, 0.17 mmol/L KCl, 0.33 mmol/L CaCl_2_). After anesthesia with tricaine/Tris pH 7.0 solution, the pronephros was isolated from zebrafish larvae as previously reported [6]. Samples were constituted by 10 pronephros each, which were stored at − 80 °C until further analysis.

For the study of the expression of *slc41a1* during zebrafish development, zebrafish were grown in E3 medium and sampled at 6, 12, 24, 48, 72, 96, and 120 hpf. For sampling, animals were anesthetized with tricaine/Tris pH 7.0 solution. Each sample was constituted of 10 embryos/larvae and was stored at − 80 °C until further analysis.

To study the regulation of *slc41a1* gene expression by dietary and water Mg^2+^ in zebrafish gills and kidney, fish Mg^2+^ balance was challenged by feeding different Mg^2+^ diets (0, 0.07, or 0.7% (*w*/*w*) Mg) or by exposure to different water Mg^2+^ concentrations (0, 0.2, 2 mmol/L) as previously reported [1]. Sampling took place 24 h after the last feeding. Fish were anesthetized in 0.1% (*v*/*v*) 2-phenoxyethanol (Sigma Chemical Co., St. Louis, USA). After anesthesia, death of animals was induced by spinal transaction and organs were collected, immediately frozen in liquid nitrogen, and stored at − 80 °C until analysis.

To study the regulation of *slc41a1* gene expression and of the zebrafish orthologs of human magnesiotropic genes expressed in the DCT (*trpm6*, *slc12a3*, *slc41a3*, *cnnm2a*, *cnnm2b*, *pro-egf*, and *fxyd2*) in 120 hpf zebrafish, zebrafish embryos were grown in different Mg^2+^-containing E3 mediums from fertilization until 120 hpf, time at which sampling took place. The different Mg^2+^-containing E3 mediums had the following electrolyte concentrations (in mmol/L): low Mg^2+^ water, 5 NaCl, 0.17 KCl, 0.33 CaCl_2_; control Mg^2+^ water, 0.33 MgSO_4_, 5 NaCl, 0.17 KCl, 0.33 CaCl_2_; high Mg^2+^ water, 25 MgSO_4_, 5 NaCl, 0.17 KCl, 0.33 CaCl_2_. At 120 hpf, zebrafish were anesthetized with tricaine/Tris pH 7.0 solution and samples (10 larvae/sample) were collected and stored at − 80 °C until further analyses.

### Knockdown of zebrafish *slc41a1*

Zebrafish eggs were obtained from natural spawning of wild-type (WT) TLF zebrafish which were bred and raised under standard conditions (28.5 °C and 14 h of light:10 h of dark cycle) in accordance with international and institutional guidelines. Two non-overlapping morpholinos (MOs) targeting *slc41a1* transcripts were used: a translation blocking MO 5′-TGATCTTTCAACCAGAGTACCATGC-3′ and a splice-site blocking MO 5′-GAAGGACTCAATGGTCTCACCTAAA-3′ (Gene Tools, Philomath, OR, USA). In parallel with these *slc41a1*-MOs, a standard mismatch MO directed (control-MO) against a human β-globin intron mutation, 5′-CCTCTTACCTCAGTTACAATTTATA (Gene Tools, Philomath, OR, USA), was used in control zebrafish. MOs were diluted in deionized, sterile water supplemented with 0.5% (*w*/*v*) phenol red and injected in a volume of 1 nL into the yolk of one- to two-cell stage embryos using a pneumatic PicoPump pv280 (World Precision Instruments, Sarasota, FL, USA). WT embryos (uninjected) were also included in the experiments to control for the effects of the injection procedure per se. After injection, embryos from the same experimental condition were placed in three Petri dishes and cultured at 28.5 °C in Mg^2+^-free E3 medium (in mmol/L: 5 NaCl, 0.17 KCl, 0.33 CaCl_2_) or normal E3 medium containing 0.33 mmol/L MgSO_4_, which was refreshed daily. Three different types of experiments were carried out: a dose-response experiment (doses of 4, 8, and 16 ng/embryo of translation or splice-site blocking *slc41a1*-MO and 16 ng/embryo control-MO, sampling at 120 hpf), a time-course experiment (doses of 16 ng/embryo of splice-site blocking MO and 16 ng/embryo control-MO, sampling at 72, 96, and 120 hpf), and an experiment to study the gene expression of *slc41a1*, *trpm6*, *slc12a3*, *slc41a3*, *cnnm2a*, *cnnm2b*, *pro-egf*, and *fxyd2* (doses of 16 ng/embryo of splice-site blocking MO and 16 ng/embryo control-MO, sampling at 120 hpf). During samplings, animals were anesthetized with tricaine/Tris pH 7.0 solution, and morphologic phenotypes were analyzed under the microscope (Leica Microsystems Ltd., Heerburgg, Germany). Two morphologic phenotypes were distinguished: normal and malformed (kidney failure features such as enlarged pericardial cavities indicating defects in glomerular filtration and/or pronephric cysts indicating defects in renal fluid flow). After phenotyping, only morphologically normal fish were sampled, each sample constituted of 10 larvae, which were stored at − 80 °C until further analyses.

### Phenotype rescue experiments in zebrafish

In vivo cRNA rescue experiments were performed with mouse WT SLC41A1 and mutant SLC41A1-p.Asp262Ala cRNAs. Constructs were subcloned into the pT7Ts expression vector, suitable for rescue experiments in zebrafish [6, 18], and cRNAs were prepared using the mMESSAGE mMACHINE Kit (Ambion, Austin, TX, USA) according to the manufacturer’s instructions. The cRNAs, in an amount of 100 pg, were co-injected with the splice-site blocking *slc41a1*-MO as described above. At 120 hpf, zebrafish were anesthetized with tricaine/Tris pH 7.0 solution, and morphologically normal zebrafish were sampled. Samples, constituted by 10 larvae/sample, were stored at − 80 °C until further analyses.

### Magnesium and calcium content measurements in zebrafish

Sample processing started by quickly washing the samples with nanopure water in order to avoid contamination of remaining water Mg^2+^ and Ca^2+^. The washing procedure was repeated twice. Fish were then dried and digested as described previously [2]. The total Mg and Ca content in each sample were determined with colorimetric assays [2, 16]. Within-run precision and accuracy were controlled by means of an internal control Precinorm (Roche Diagnostics, Mannheim, Germany). Furthermore, samples were normalized by protein content, which was determined with the Pierce BCA protein assay kit (Pierce Biotechnology, Rockford, IL, USA).

### RNA isolation and cDNA synthesis

RNA was isolated from zebrafish adult tissues, the pronephros, and total embryos/larvae using TRIzol reagent (Invitrogen, Carlsbad, CA, USA) according to the manufacturer’s instructions, in which glycogen (Fermentas GmbH, St. Leon-Rot, Germany) was used in order to maximize the RNA recovery. One microgram of RNA (200 ng in the case of the pronephric samples) was subjected to DNase treatment to prevent genomic DNA contamination and subsequently used to perform the reverse transcriptase reaction.

### Real-time quantitative polymerase chain reaction

Changes in target genes mRNA levels were determined by relative real-time quantitative polymerase chain reaction (RT-qPCR) as previously described [1] and in accordance with the MIQUE guidelines [4] with a CFX96™ Real-Time PCR Detection System (Bio-Rad Laboratories, Hercules, CA) using iQ™ SYBR Green Supermix (Bio Rad) detection of single PCR product accumulation. Gene expression levels were normalized to the expression levels of the standard zebrafish-specific reference *elongation factor-1α* (*elf1α*) [1, 22]. Relative mRNA expression was analyzed using the Livak method (2^−ΔΔCt^). Primer sequences are shown in Table 2.

**Table 2:** Primer sequences for gene expression and to determine the knockdown mechanism and efficacy of the splice-site blocking MO

Gene	Sequence		Use
*slc41a1*	5′-CACCGGGACGGAAAGGATAG-3′	FW1	Knockdown mechanism (PCR)
*slc41a1*	5′-TGGATGCGATGATAACCCCG-3′	RV1	Knockdown mechanism (PCR)
*slc41a1*	5′-GACATGGTGGTCCTCGACTG-3′	FW2	Knockdown mechanism (PCR)
*slc41a1*	5′-CCAACCTTCCTGGATGCGAT-3′	RV2	Knockdown mechanism (PCR)
*slc41a1*	5′-ATGGGAAACCTCGCGCTAAT-3′	FW3	Knockdown mechanism (PCR)
*slc41a1*	5′-ATGGCGATGATGACAGGCTC-3′	RV3	Knockdown mechanism (PCR)
*slc41a1*	5′-CCGCATTCATCGCCTCTTTG-3′	FW	RT-qPCR quantification of non-spliced (functional) *slc41a1* transcripts
*slc41a1*	5′-CAATAGGGGTGGCCACGTT-3′	RV	RT-qPCR quantification of non-spliced (functional) *slc41a1* transcripts
*slc41a1*	5′-TCCGGTCATTAACGGTGTGGGTG-3′	FW	RT-qPCR quantification of total *slc41a1* transcripts
*slc41a1*	5′-GCAGGGCGTTCATGTGCAGG-3′	RV	RT-qPCR quantification of total *slc41a1* transcripts
*trpm6*	5′-GCTGCAGCACACCAGCCTCAG-3′	FW	RT-qPCR
*trpm6*	5′-GCACATACTTAGCACGACACGCACG-3′	RV	RT-qPCR
*slc12a3*	5′-ACCATTGGCTCCTGTGTGGTGC-3′	FW	RT-qPCR
*slc12a3*	5′-TGCAGCCCACTCCCAGACACT-3′	RV	RT-qPCR
*slc41a3*	5′-TGTTGGTGTACGGAACGGAC-3′	FW	RT-qPCR
*slc41a3*	5′-CTTCTTGCGACCACCTCCTT-3′	RV	RT-qPCR
*cnnm2a*	5′-GATGAAGGCGGACGCGGACT-3′	FW	RT-qPCR
*cnnm2a*	5′-TGCGGTCCATTGCTCTGCCA-3′	RV	RT-qPCR
*cnnm2b*	5′-AGTGCGATCAGTTTCAGCCGC-3′	FW	RT-qPCR
*cnnm2b*	5′-GCTTCATCTCTGCTGCGCGT-3′	RV	RT-qPCR
*pro-egf*	5′-CCAGGCCGGTGGGTTTGTGA-3′	FW	RT-qPCR
*pro-egf*	5′-TGGCAAACAACAGGTCCGCTGG-3′	RV	RT-qPCR
*fxyd2*	5′-AAGATGGGAGTGGAAAGTCCTGAGC-3′	FW	RT-qPCR
*fxyd2*	5′-AAACAGCTGCGAAGATCAGTCCTCC-3′	RV	RT-qPCR
*elf1α*	5′-GAGGCCAGCTCAAACATGGGC-3′	FW	RT-qPCR
*elf1α*	5′-AGGGCATCAAGAAGAGTAGTACCGC-3′	RV	RT-qPCR

*FW* forward primer, *RV* reverse primer

### Knockdown mechanism and efficacy of the splice-site blocking *slc41a1*-MO

To determine the knockdown mechanism induced by the splice-site blocking *slc41a1*-MO, cDNA from 120 hpf control (injected with 16 ng control-MO) and morphant (injected with 16 ng splice-site blocking *slc41a1*-MO) larvae was used for PCR analysis. Three different primer pairs flanking exon 5 were used (Table 2). Amplicons thus obtained were subjected to electrophoresis in 1.5% (*w*/*v*) agarose gel. The efficacy of the splice-site blocking MO targeting exon 5 (145 bp) in the *slc41a1* gene was measured by RT-qPCR following the methods described above. The specific primers used are shown in Table 2. The forward primer was designed to hybridize exon 5, the exon targeted by the splice-site blocking *slc41a1*-MO. Thus, the resulting amplicon quantification reflects the expression levels of the functional, non-spliced *slc41a1*. In addition, *slc41a1* expression of the total *slc41a1* transcript, which entails non-spliced and aberrantly spliced variants, was measured by RT-qPCR (primers used are shown in Table 2).

### Cell culture

HEK293 cells were grown in Dulbecco’s modified Eagle’s medium (DMEM, Bio Whittaker-Europe, Verviers, Belgium) containing 10% (*v*/*v)* fetal calf serum (PAA, Liz, Austria), 2 mmol/L L-glutamine, and 10 μg/mL non-essential amino acids, at 37 °C in a humidity-controlled incubator with 5% (*v*/*v*) CO_2_. The cells were transiently transfected with the respective DNA constructs using Lipofectamine 2000 (Invitrogen, Breda, The Netherlands) at 1:2 DNA:Lipofectamine ratio for 48 h unless otherwise stated.

Madin-Darby kidney (MDCKI) cells were cultured in DMEM with 5% (*v*/*v*) fetal calf serum (PAA, Liz, Austria), 2 mmol/L L-glutamine, ciproxin, and 10 μg/mL non-essential amino acids, at 37 °C in a humidity-controlled incubator with 5% (*v*/*v*) CO_2_. The cells were stably transfected with SLC41A1 using Metafectene pro (Biontex) in 1:3 DNA:Metafectene ratio. After 48 h, transfected cells were placed on selection medium containing 0.8 mg/mL G418 and 0.15 mg/mL hygromycin for 14 days.

### Magnesium transport assays

HEK293 cells were seeded in 12-well plates (0.5 × 10^6^ cells/12 wells) and transfected with empty (mock), WT, and mutant SLC41A1 constructs for 48 h and seeded on poly-L-lysine (Sigma)-coated 12-well plates. For extrusion experiments, the cells were transferred to Mg^2+^-free medium supplemented with 1 mmol/L ^25^Mg^2+^ during the last 24 h (purity ± 98%, Cortecnet, Voisins le Bretonneux, France). Before the start of the experiment, cells were washed three times in basic uptake buffer (in mmol/L: 125 NaCl, 5 KCl, 0.5 CaCl_2_, 0.5 Na_2_HPO_4_, 0.5 Na_2_SO_4_, 15 HEPES/NaOH pH 7.5) and subsequently placed in basic uptake buffer with 0.5 mmol/L Mg^2+^ (Sigma, containing ± 10% ^25^Mg^2+^) for 5, 10, or 15 min. For experiments using NMDG, NaCl was replaced in the buffer by NMDG (pH 7.4 with HCl), and in the Cl-free experiments, NaCl, KCl, and CaCl_2_ were replaced by Na-gluconate, K-gluconate, and Ca-gluconate (pH 7.4 with NaOH). The buffer was removed and subjected to inductively coupled plasma mass spectrometry (ICP-MS) analysis. Uptake experiments were performed as described previously [2]. Naturally occurring ^25^Mg content of the cells was determined at time point 0 and was subtracted from the other time points when calculating net fluxes. In the experiments using pharmacological inhibition, 100 μM quinidine, 100 μM ouabain, and 100 μM 2-APB were added 30 min before the start of the uptake. The coefficient of variation (CV) for intra-assay and inter-assay variability has been calculated to determine the variability of our assay. The intra-assay CV is 4% and the inter-assay CV is 12%.

### Cell surface biotinylation

HEK293 cells were transfected with WT and mutant SLC41A1 constructs for 48 h. Cell surface proteins were biotinylated as described previously [2]. Protein lysates were subjected to SDS-PAGE and immunoblots were incubated using mouse anti-HA (1:5000; Cell Signaling, Danvers, MA, USA).

### Western blot

HEK293 cells were transfected for 48 h with WT and mutant SLC41A1 constructs. Protein lysates were denatured in Laemmli containing 100 mmol/L DTT for 30 min at 37 °C and subsequently subjected to SDS-PAGE. Then, immunoblots were incubated with a mouse anti-HA (Roche, high affinity 3F10, 1:5000) primary antibody and peroxidase-conjugated sheep anti-mouse secondary antibodies (Jackson Immunoresearch, 1:10,000).

### Immunocytochemistry

SLC41A1-expressing MDCKI cells were seeded on 0.4 μm transwell filters (Corning Costar) and cultured for 7 days. Subsequently, the cells were rinsed with PBS and fixed for 30 min with 4% (*w*/*v*) paraformaldehyde solution in PBS, permeabilized in 0.3% (*v*/*v*) Triton in PBS for 15 min, and quenched for 15 min in 50 mM NH_4_Cl. After blocking with goat serum (16% (*v*/*v*) for 30 min), cells were incubated with primary antibody rat anti-HA (Roche Applied Science, 1:250) or anti-E-Cadherin (Sigma, 1:250) for 60 min. After rinses in PBS, cells were incubated with Alexa Fluor 488-conjugated donkey anti-rat IgG or Alexa Fluor 594-conjugated donkey anti-rabbit IgG secondary antibodies (Jackson, 1:300) and DAPI for 45 min. After PBS and ethanol rinses, the cells were mounted on slides using Mowiol. Fluorescence microscopy was performed with a Zeiss inverted microscope, and images were taken with ImageJ software.

### Statistics

All results are depicted as mean ± standard error of the mean (S.E.M.). Statistical analyses were conducted by one-way ANOVA. Tukey’s post-test was used to identify significantly different groups. When only two experimental groups were present (e.g., analysis of *slc41a1* expression in the pronephros), an unpaired Student’s *t* test was used. Statistical significance was accepted at *P* ˂ 0.05.

## Results

### Gene expression of *slc41a1* is Mg^2+^ dependent

To examine the involvement of SLC41A1 in Mg^2+^ homeostasis, the zebrafish model was used. The zebrafish is a versatile model to study electrolyte handling in mammals, as its nephron segmentation patterns and ion transport functions are similar to those present in the mammalian nephron [2, 6, 12, 19, 30]. The zebrafish ortholog of mammalian SLC41A1 is highly conserved: 77% of amino acid (AA) identity compared to its human counterpart (Online Resource 1). In general, the 10 hydrophobic transmembrane domains (TMD) are well conserved between zebrafish Slc41a1 and human, mouse, or rat SLC41A1 (Online Resource 1). The two motifs PX_6_GN and P(D/A)X_4_PX_6_D, which form the Mg^2+^ selective pore [21], are present in the zebrafish Slc41a1 protein and display an identity of 100% with respect to the human, mouse, and rat SLC41A1 (Online Resource 1).

**Fig. 1:**
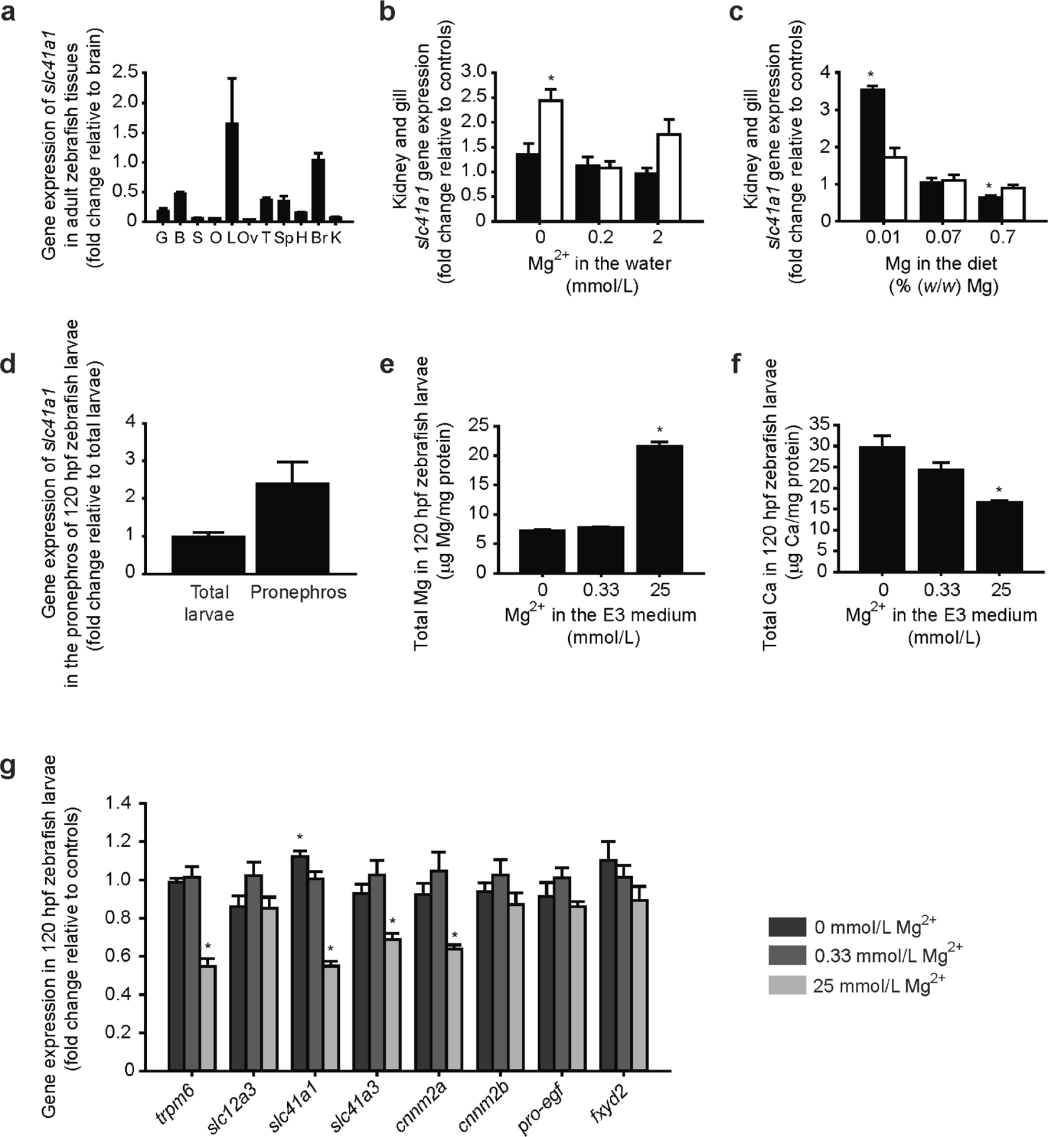
The gene *slc41a1* is ubiquitously expressed in zebrafish tissues and its expression is regulated by dietary and/or water Mg^2+^ in adult gills and kidney and in total larvae. **a** Tissue and organ distribution of zebrafish *slc41a1* gene expression (*n* = 6, except for the ovary and testis where *n* = 3). **b**, **c** Expression levels of *slc41a1* in the kidney (black bars) and gills (white bars) of adult zebrafish exposed to low Mg^2+^ water, control Mg^2+^ water, and high Mg^2+^ water with a nominal concentration of Mg^2+^ of 0, 0.2, and 2 mmol/L respectively (**b**) or fed a low Mg^2+^ diet, a control Mg^2+^ diet, and a high Mg^2+^ diet with a nominal concentration of Mg of 0, 0.07, and 0.7% (*w*/*w*) Mg respectively (**c**) (*n* = 8–9). **d***slc41a1* gene expression in zebrafish pronephric tissue and in total larvae at 120 hpf (*n* = 3). **e**, **f** Total Mg (**e**) and Ca (**f**) content in 120 hpf zebrafish grown in E3 media with different Mg^2+^ concentrations, namely 0 mmol/L Mg^2+^ (low Mg^2+^ water), 0.33 mmol/L Mg^2+^ (control Mg^2+^ water), and 25 mmol/L Mg^2+^ (high Mg^2+^ water) (*n* = 10). **g** Gene expression of zebrafish orthologs of mammalian DCT-specific genes and of *slc41a1* in zebrafish larvae (120 hpf) grown in E3 media with different Mg^2+^ concentrations, namely 0 mmol/L Mg^2+^ (low Mg^2+^ water), 0.33 mmol/L Mg^2+^ (control Mg^2+^ water), and 25 mmol/L Mg^2+^ (high Mg^2+^ water) (*n* = 9–10). **a**–**g** Data are presented as mean ± S.E.M. **a**–**d**, **g** Gene expression levels were assessed by RT-qPCR and normalized against the housekeeping gene *elf1α*. Data were calculated using the Livak method (2^−ΔΔCt^). **b**, **c**, **e**–**g** Asterisks indicate significant differences between control and experimental groups exposed to low or high Mg^2+^ water, or fed a low or high Mg^2+^ diet (*P < 0.05)

Similar to mammals [10], the zebrafish *slc41a1* gene is ubiquitously expressed in adult tissues (Fig. 1a). The gene expression of *slc41a1* in the kidney and gills of adult zebrafish is regulated by dietary and water Mg^2+^, respectively (Fig. 1b, c). In zebrafish larvae (120 hpf), *slc41a1* is expressed in the pronephros (Fig. 1d). Growth of zebrafish in different Mg^2+^-containing E3 mediums evoke distinct total Mg and Ca content patterns in 120 hpf zebrafish larvae (Fig. 1e, f). Importantly, gene expression of *slc41a1* is regulated by Mg^2+^ in these larvae (120 hpf), similarly to the orthologs of mammalian DCT-specific genes such as *trpm6*, *slc41a3*, and *cnnm2a* (Fig. 1g).

### Loss of Slc41a1 function causes Mg^2+^ deficiency in zebrafish

**Fig. 2:**
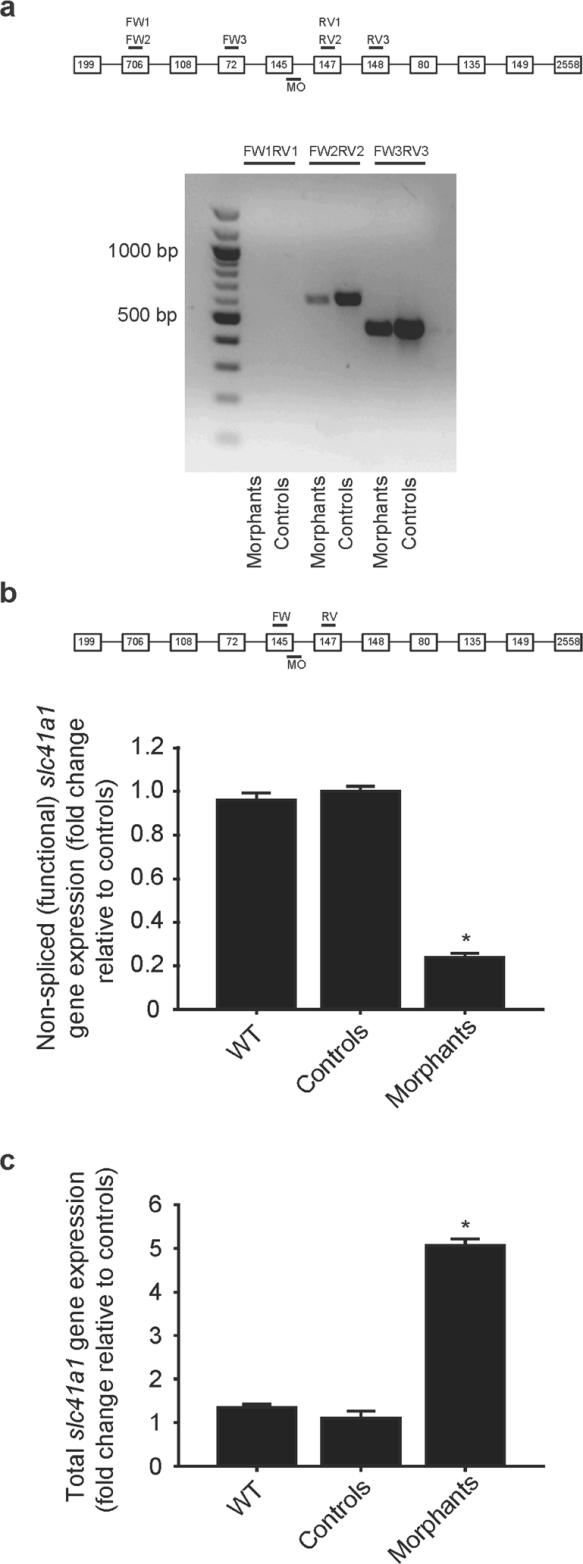
Knockdown mechanism and efficacy of the splice-site blocking *slc41a1*-MO. **a** Gel electrophoresis image showing knockdown of *slc41a1* gene expression following injection of the splice-site blocking *slc41a1*-MO (morphants, dose of 16 ng *slc41a1*-MO/embryo) or control-MO (controls, dose of 16 ng control-MO/embryo) in 120 hpf zebrafish larvae. Three different primer combinations were used: FW1RV1, FW2RV2, and FW3RV3. Under our PCR conditions, FW1RV1 did not yield any amplification. Bands showing correctly spliced *slc41a1* mRNA are shown. Aberrantly spliced *slc41a1* mRNA was not detected, indicating that the splice-site blocking *slc41a1*-MO probably evoked (partial or total) intron inclusions. Shown on the left are the sizes (in bp) of the major bands of the DNA ladder. The expected amplicon size of the correctly spliced *slc41a1* mRNA using FW2 and RV2 primers is of 602 bp, and when using FW3 and RV3 is of 452 bp. **b** Quantification of the knockdown evoked by the splice-site blocking *slc41a1*-MO (16 ng *slc41a1*-MO/embryo) by RT-qPCR using specific primers that discriminate for the correctly spliced (functional) *slc41a1* mRNA. **c** Quantification of the expression levels of total *slc41a1* transcripts in WT, control (injected with 16 ng control-MO/embryo), and *slc41a1* morphant (injected with 16 ng *slc41a1*-MO/embryo) zebrafish. The FW primer used binds the exon 8–exon 9 junction while the RV primer used binds exon 9 (135 bp). **b**, **c** Fish selected for analysis were morphologically normal, grown in Mg^2+^-free E3 medium, and sampled at 120 hpf. Data were normalized against the housekeeping gene *elf1α* and calculated using the Livak method (2^−ΔΔCt^). Data are presented as mean ± S.E.M. (*n* = 9–10). Asterisks indicate significant differences between the control and the *slc41a1*-knockdown group (*P < 0.05)

**Fig. 3:**
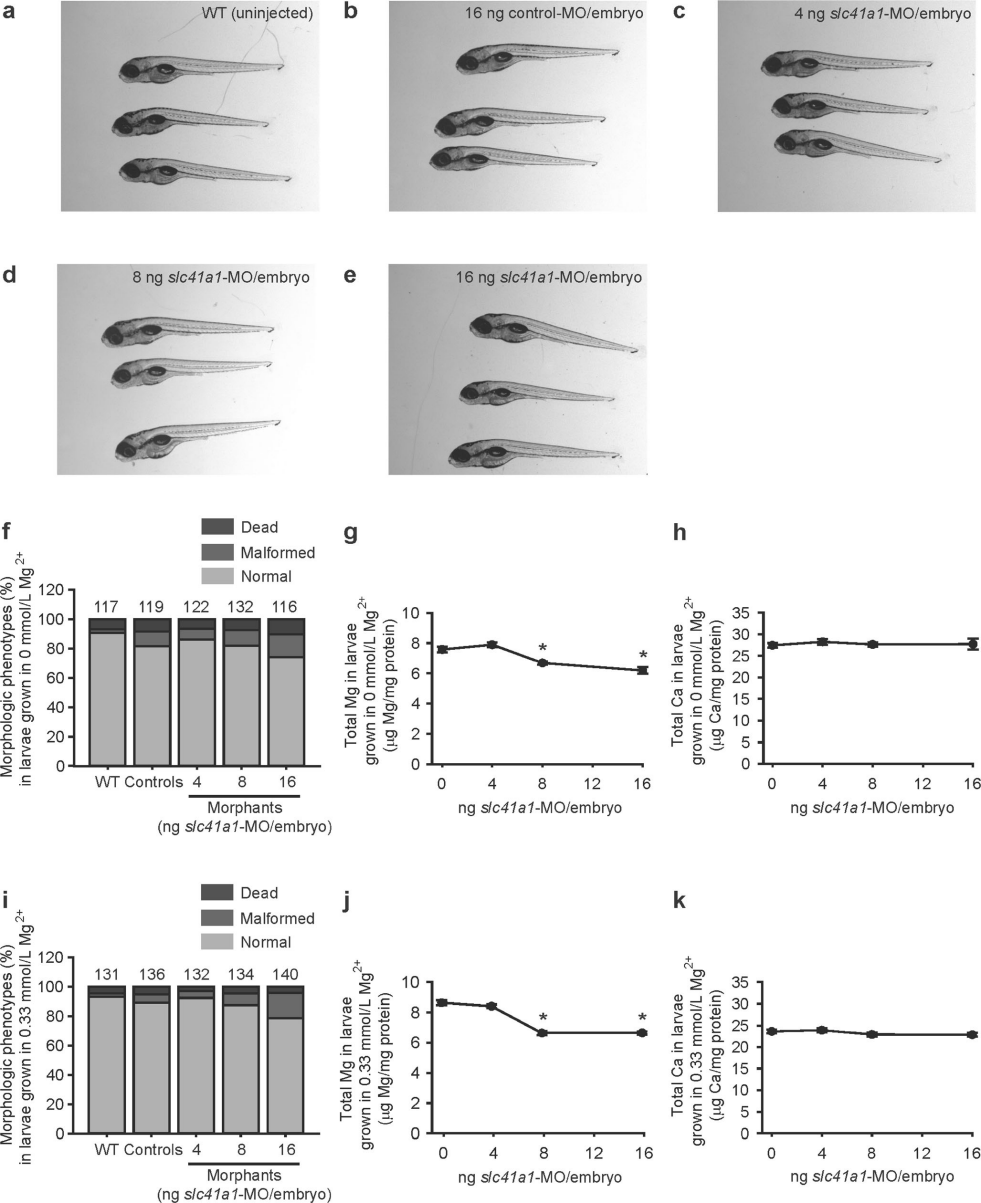
Slc41a1 knockdown evokes renal Mg^2+^ wasting in zebrafish. **a**–**e** Representative images of the normal morphologic phenotypes obtained in untreated (WT) 120 hpf zebrafish grown in Mg^2+^-free E3 medium and after injection of 16 ng control-MO/embryo or 4–16 ng *slc41a1*-MO/embryo (splice-site blocking MO). **f** Distribution of morphologic phenotypes in zebrafish larvae (120 hpf) untreated (WT) or after injection with different doses of *slc41a1*-MO (splice-site blocking MO) or control-MO. Fish were grown in Mg^2+^-free E3 medium. Numbers on top of the bars indicate the number of animals in each experimental condition. **g**, **h** Total Mg (**g**) and Ca (**h**) content in morphologically normal 120 hpf zebrafish grown in Mg^2+^-free E3 medium after injection with different doses of *slc41a1*-MO (splice-site blocking MO); the dose of zero represents injection with 16 ng control-MO/embryo (*n* = 10). **i** Distribution of morphologic phenotypes in zebrafish larvae (120 hpf) untreated (WT) or after injection with different doses of *slc41a1*-MO (splice-site blocking MO) or control-MO. Fish were grown in E3 medium (0.33 mmol/L Mg^2+^). Numbers on top of the bars indicate the number of animals in each experimental condition. **j**, **k** Total Mg (**j**) and Ca (**k**) content in morphologically normal 120 hpf zebrafish grown in E3 medium (0.33 mmol/L Mg^2+^) after injection with different doses of *slc41a1*-MO (splice-site blocking MO); the dose of zero represents injection with 16 ng control-MO/embryo (*n* = 10). **g**, **h**, **j**, **k** Data are presented as mean ± S.E.M. Asterisks indicate significant differences between control and experimental groups (*P < 0.05)

Knockdown of *slc41a1* by a splice-site blocking MO in zebrafish grown in a Mg^2+^-free E3 medium resulted in a downregulation of functional (non-spliced) *slc41a1* transcripts (Fig. 2b). In addition, the morphology between *slc41a1* morphants and controls was identical (Fig. 3a–f). However, the total Mg content was significantly decreased in *slc41a1* morphants compared to controls while the total Ca content remained unchanged (Fig. 3g, h). This result is not secondary to any morphologic/developmental phenotype since total Mg and Ca levels were determined in morphologically normal *slc41a1* morphants and controls.

**Fig. 4:**
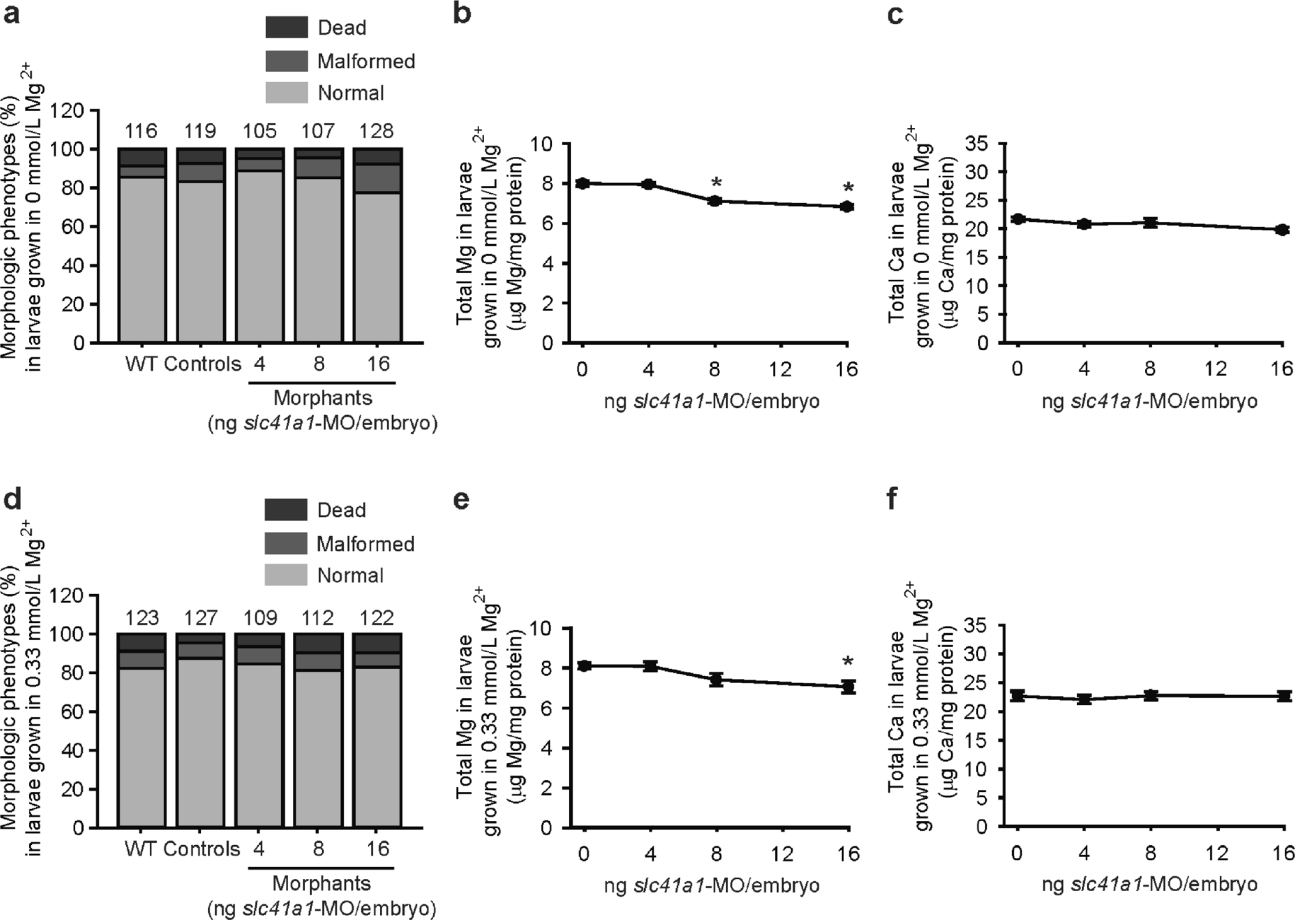
Renal Mg^2+^ wasting in *slc41a1* morphants is not an off-target effect of the *slc41a1*-knockdown approach used. **a** Distribution of morphologic phenotypes in zebrafish larvae (120 hpf) untreated (WT) or after injection with different doses of *slc41a1*-MO (translation-blocking) or control-MO. Fish were grown in Mg^2+^-free E3 medium. Numbers on top of the bars indicate the number of animals in each experimental condition. **b**, **c** Total Mg (**b**) and Ca (**c**) content in morphologically normal 120 hpf zebrafish grown in Mg^2+^-free E3 medium after injection with different doses of *slc41a1*-MO (translation-blocking); the dose of zero represents injection with 16 ng control-MO/embryo (*n* = 10). **d** Distribution of morphological phenotypes in zebrafish larvae (120 hpf) untreated (WT) or after injection with different doses of *slc41a1*-MO (translation-blocking) or control-MO. Fish were grown in E3 medium (0.33 mmol/L Mg^2+^). Numbers on top of the bars indicate the number of animals in each experimental condition. **e**, **f** Total Mg (**e**) and Ca (**f**) content in morphologically normal 120 hpf zebrafish grown in E3 medium (0.33 mmol/L Mg^2+^) after injection with different doses of *slc41a1*-MO (translation-blocking); the dose of zero represents injection with 16 ng control-MO/embryo (n = 10). **b**, **c**, **e**, **f** Data are presented as mean ± S.E.M. Asterisks indicate significant differences between control and experimental groups (*P < 0.05)

When *slc41a1* morphant larvae were grown in Mg^2+^-containing E3 medium (0.33 mM Mg^2+^), the morphology of *slc41a1* morphants was also comparable to controls (Fig. 3i), and the total Mg content was not restored in morphologically normal zebrafish *slc41a1* morphants to the levels observed in morphologically normal control larvae (Fig. 3j, k). The specificity of the decreased Mg content observed when knocking down *slc41a1* in zebrafish with the splice-site blocking MO was confirmed by repeating the experiments with a second non-overlapping oligo, a translation blocking MO: similar results were obtained (Fig. 4).

**Fig. 5:**
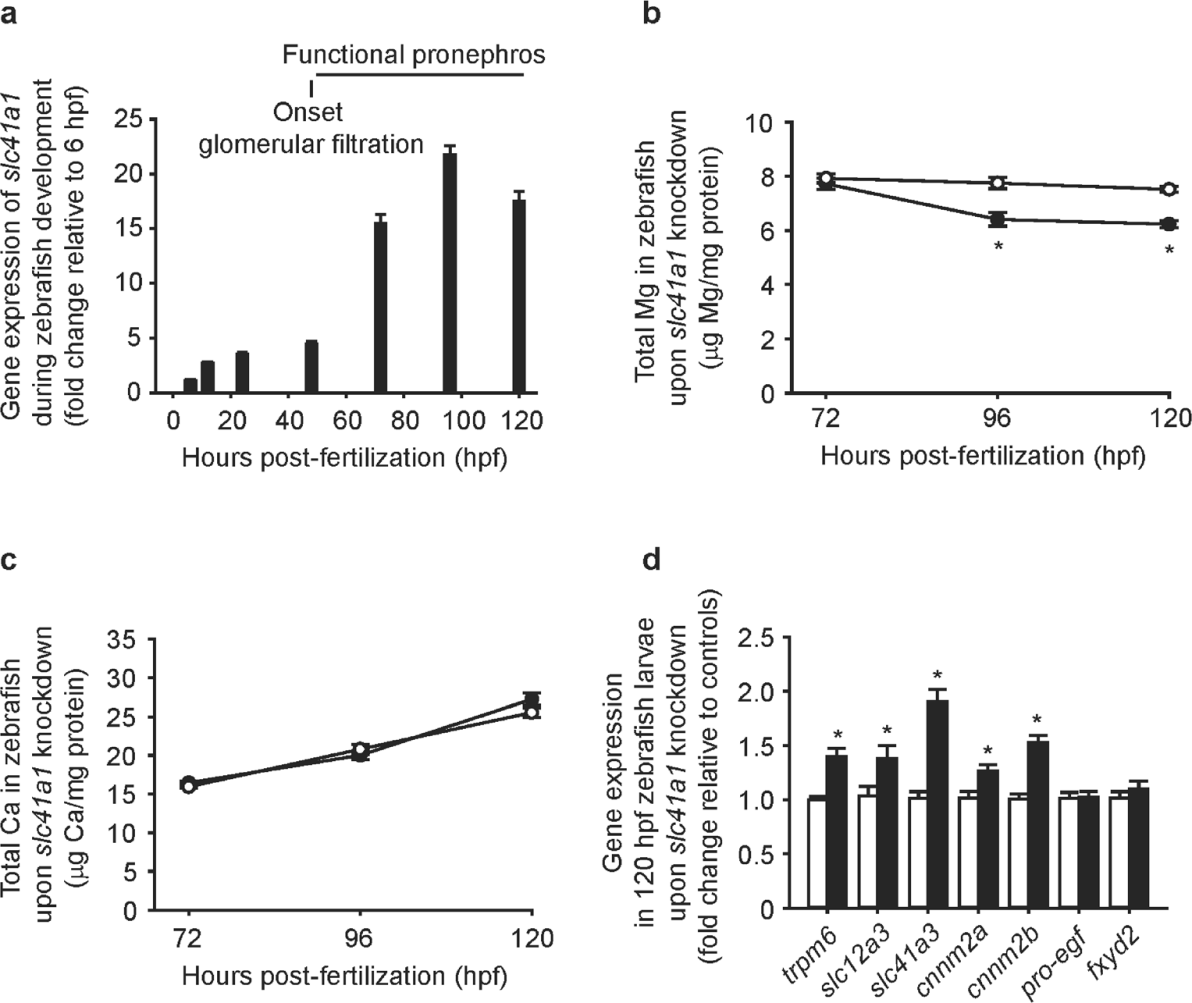
Renal Mg^2+^ wasting in Slc41a1 knockdown correlates with pronephros maturation and evokes DCT-specific compensatory mechanisms in zebrafish. **a***slc41a1* gene expression during zebrafish development (*n* = 6). **b**, **c** Time course of the total Mg (**b**) and Ca (**c**) content in morphologically normal zebrafish grown in Mg^2+^-free E3 medium after injection of 16 ng control-MO/embryo (white circles) or 16 ng *slc41a1*-MO/embryo (splice-site blocking MO, black circles) (*n* = 10). **d** Gene expression of the orthologs of mammalian DCT-specific genes in morphologically normal 120 hpf zebrafish grown in Mg^2+^-free E3 medium after injection of 16 ng *slc41a1*-MO/embryo (splice-site blocking, black bars) relative to 16 ng control-MO/embryo (white bars) (*n* = 9–10). **a**–**d** Data are presented as mean ± S.E.M. **a**, **b** Gene expression levels were assessed by RT-qPCR and normalized against the housekeeping gene *elf1α*. Data were calculated using the Livak method (2^−ΔΔCt^). **b**–**d** Data are presented as mean ± S.E.M. Asterisks indicate significant differences between control and experimental groups in the same day of sampling (*P < 0.05)

Importantly, the disturbances in the total Mg content in morphologically normal zebrafish *slc41a1* morphants grown in Mg^2+^-free E3 medium were detectable only from 96 hpf, a time point in which the pronephros is fully functional [31] and at which *slc41a1* shows its highest gene expression levels in zebrafish (Fig. 5a, b). Conversely, total Ca levels were comparable between *slc41a1* morphants and controls during development (Fig. 5c). Moreover, potential DCT compensatory mechanisms to the decreased total Mg levels were observed in *slc41a1* morphants and were illustrated by an upregulation of *trpm6*, *slc12a3*, *slc41a3*, *cnnm2a*, and *cnnm2b* gene expression (Fig. 5d). Importantly, the expression of total *slc41a1* transcripts was also upregulated in *slc41a1* morphants (Fig. 2c), further corroborating the regulation of *slc41a1* expression by Mg^2+^.

### Mammalian SLC41A1 and zebrafish Slc41a1 are functionally equivalent

**Fig. 6:**
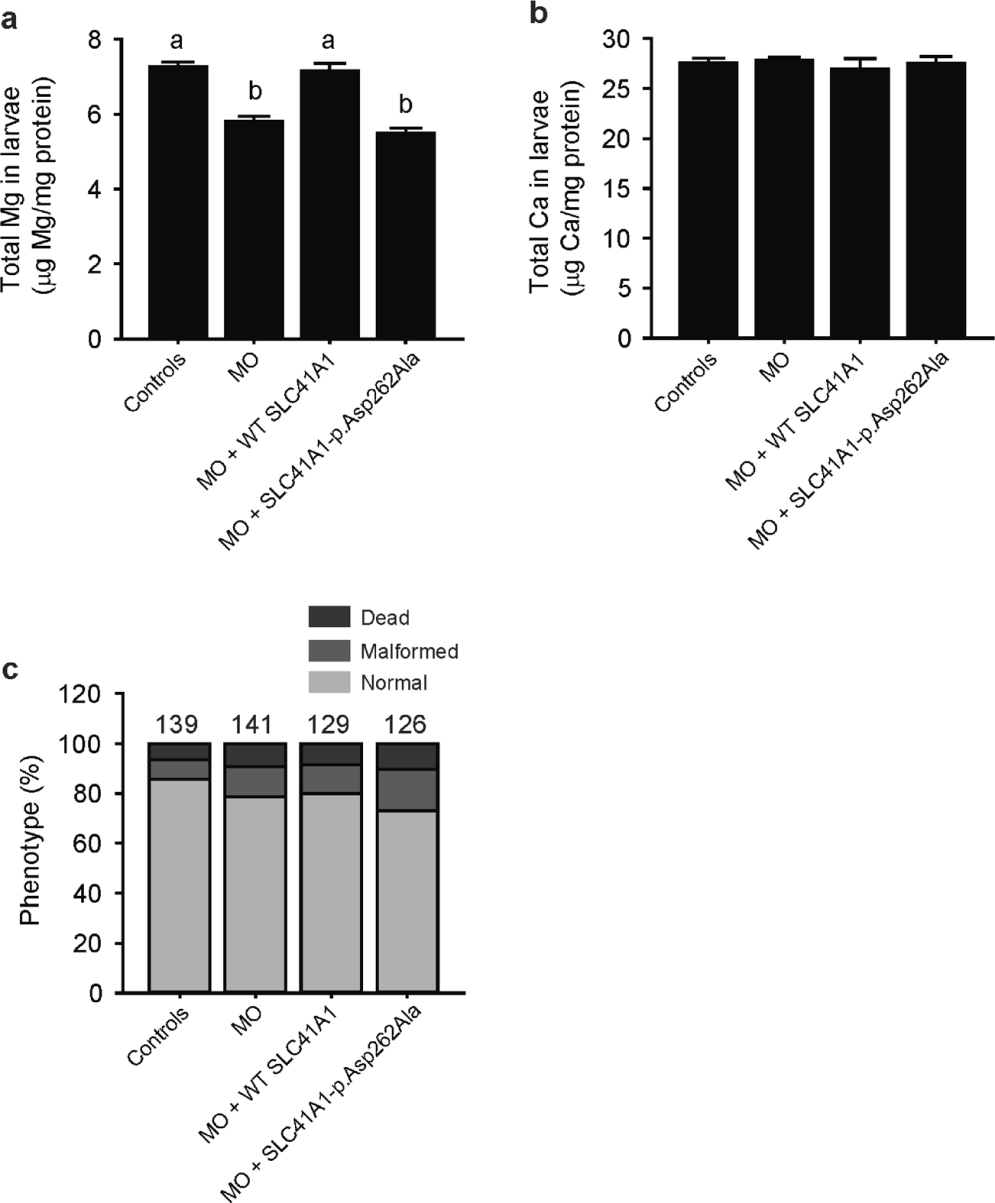
Renal Mg^2+^ wasting in zebrafish *slc41a1* morphants is rescued by mammalian *Slc41a1* expression. **a** Total Mg levels in morphologically normal 120 hpf zebrafish grown in Mg^2+^-free E3 medium after injection of 16 ng control-MO/embryo, of 16 ng *slc41a1*-MO/embryo (splice-site blocking MO), and after co-injection of 16 ng *slc41a1*-MO/embryo with 100 pg/embryo of WT SLC41A1 or SLC41A1-pAsp262Ala cRNA (*n* = 9–10). **b** Total Ca levels in morphologically normal 120 hpf zebrafish grown in Mg^2+^-free E3 medium after injection of 16 ng control-MO/embryo, of 16 ng *slc41a1*-MO/embryo (splice-site blocking MO), or co-injection of 16 ng *slc41a1*-MO/embryo with 100 pg/embryo of WT SLC41A1 or SLC41A1-pAsp262Ala cRNA (*n* = 9–10). **c** Distribution of morphologic phenotypes in zebrafish larvae (120 hpf) grown in Mg^2+^-free E3 medium after injection of 16 ng control-MO/embryo, of 16 ng *slc41a1*-MO/embryo (splice-site blocking MO), and after co-injection of 16 ng *slc41a1*-MO/embryo with 100 pg/embryo of WT SLC41A1 or SLC41A1-pAsp262Ala cRNA. **a**, b Data are presented as mean ± S.E.M. Different letters indicate significant differences between groups (*P* < 0.05)

The functional equivalency between zebrafish Slc41a1 and mammalian SLC41A1 was demonstrated by the fact that co-injection of the splice-site blocking MO against zebrafish *slc41a1* with mouse WT *Slc41a1* cRNA resulted in total Mg levels in 120 hpf zebrafish comparable to controls (Fig. 6a). In addition, these results further proved the specificity of the knockdown approach used in the zebrafish. Importantly, expression in *slc41a1* morphants of the mouse mutant SLC41A1-p.Asp262Ala, homologous with the pore MgtE-p.Asp432Ala mutant that abolishes MgtE Mg^2+^ transport in *Thermus thermophilus* [15], did not result in similar total Mg levels between *slc41a1* morphants and controls (Fig. 6a). No changes in total Ca levels were observed (Fig. 6b). As in previous experiments, total Mg and Ca levels were measured only in morphologically normal *slc41a1* morphants and controls. Similar proportions of morphologic phenotypes were observed in all experimental conditions tested (Fig. 6c).

### SLC41A1 facilitates Mg^2+^ extrusion in HEK293 cells

**Fig. 7:**
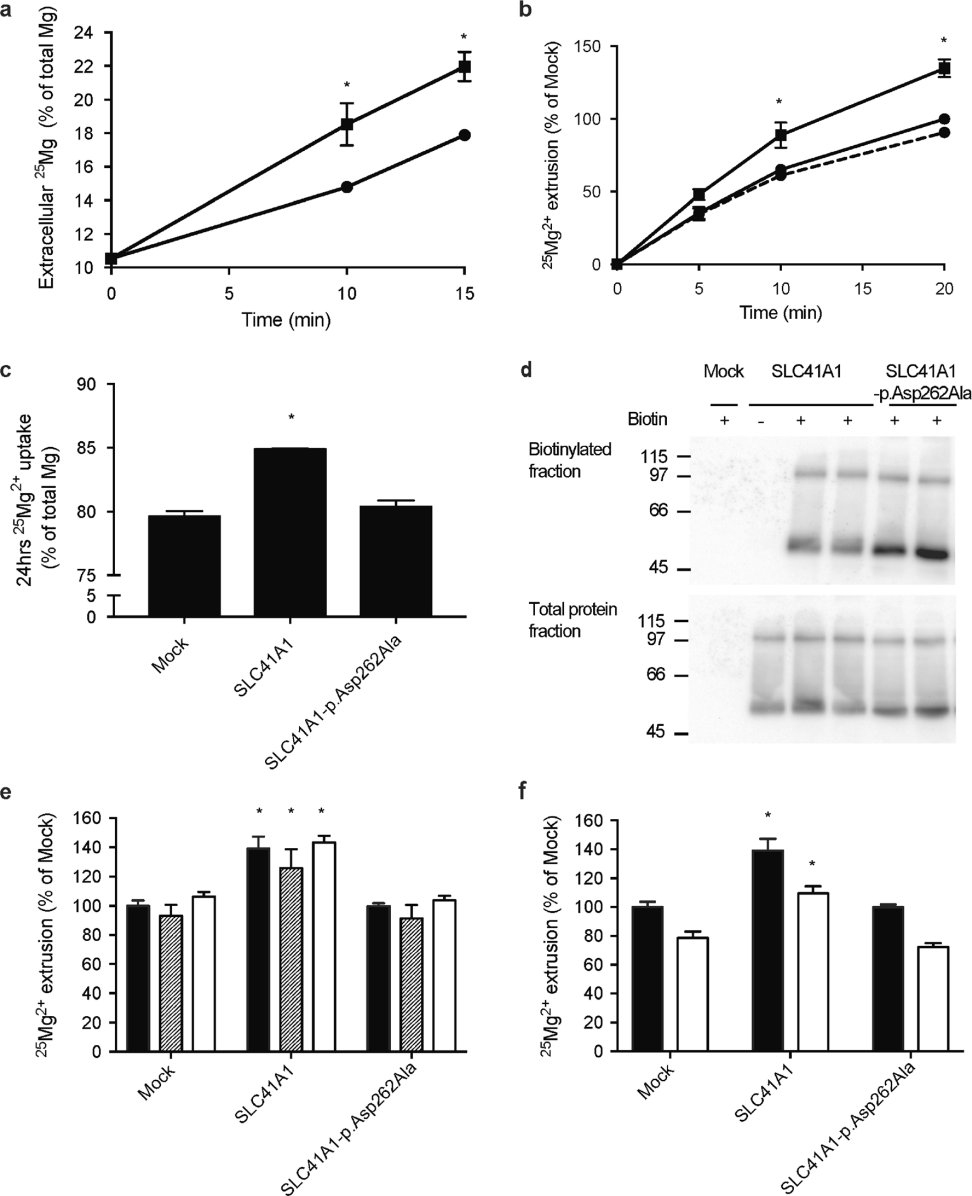
SLC41A1 mediates cellular Mg^2+^ extrusion. **a** Time curve of ^25^Mg^2+^ extrusion in mock and SLC41A1-transfected HEK293 cells, indicated as % of the total extracellular Mg^2+^. **b** Time curve of ^25^Mg^2+^ extrusion in mock, SLC41A1, and SLC41A1-p.Asp262Ala-transfected HEK293 cells compared to the mock condition. **c** HEK293 cells were transfected with mock, SLC41A1, or SLC41A1-p.Asp262Ala, and loaded for 24 h with ^25^Mg^2+^. Subsequently, intracellular ^25^Mg^2+^ content is measured. **d** Immunoblots showing comparable membrane expression between WT and p.Asp262Ala SLC41A1 proteins (upper blot). The lower blot shows a SLC41A1 expression control. The blots show a representative experiment of three independent experiments. **e**^25^Mg^2+^ extrusion after 15 min in mock, SLC41A1, and SLC41A1-p.Asp262Ala-transfected HEK293 cells in basic uptake buffer (black bars), NMDG-Cl containing uptake buffer (striped bars), and Na-gluconate containing uptake buffer (white bars). All results are relative to the mock condition in the basic uptake buffer. **f**^25^Mg^2+^ extrusion after 15 min in mock, SLC41A1, and SLC41A1-p.Asp262Ala-transfected HEK293 cells in basic uptake buffer (black bars) and supplemented with 100 μM ouabain (white bars). All results are relative to the mock condition in the basic uptake buffer. All graphs show the mean of three independent experiments ± SEM, *P < 0.05 compared to mock

SLC41A1 has been proposed to function as basolateral Na^+^-Mg^2+^ exchanger [20]. To examine the Mg^2+^ transport capacities of SLC41A1, HEK293 cells were loaded for 24 h with ^25^Mg^2+^; subsequently, extrusion was monitored for 15 min. ^25^Mg^2+^ extrusion was increased in SLC41A1-expressing cells compared to mock-transfected cells (Fig. 7a). To show that the Mg^2+^ extrusion is facilitated by SLC41A1, the Mg^2+^-selective residue of the putative pore forming domain of SLC41A1 was mutated (p.Asp262Ala) [21]. Indeed, Mg^2+^ extrusion in cells expressing SLC41A1-p.Asp262Ala was comparable to mock (Fig. 7b). Cells expressing SLC41A1 had marginally, but significantly, higher intracellular ^25^Mg levels after 24 h loading (Fig. 7c). Plasma membrane expression of WT and mutant SLC41A1 proteins was comparable, as shown by cell surface biotinylation (Fig. 7d). To examine whether the ^25^Mg^2+^ extrusion depends on Na^+^ or Cl^−^, the NaCl in the basic buffer was replaced for NMDG-Cl or Na-gluconate, respectively. Interestingly, SLC41A1-dependent ^25^Mg^2+^ extrusion was present in all conditions (Fig. 7e). To exclude a potential role of the membrane potential on SLC41A1-mediated ^25^Mg^2+^ extrusion, cells were incubated with ouabain, an inhibitor of the Na^+^-K^+^-ATPase. Although ouabain reduced ^25^Mg^2+^ extrusion in both mock and SLC41A1 expressing cells, it did not change the SLC41A1-mediated extrusion.

### SLC41A1-mediated Mg^2+^ transport is independent of Na^+^

**Fig. 8:**
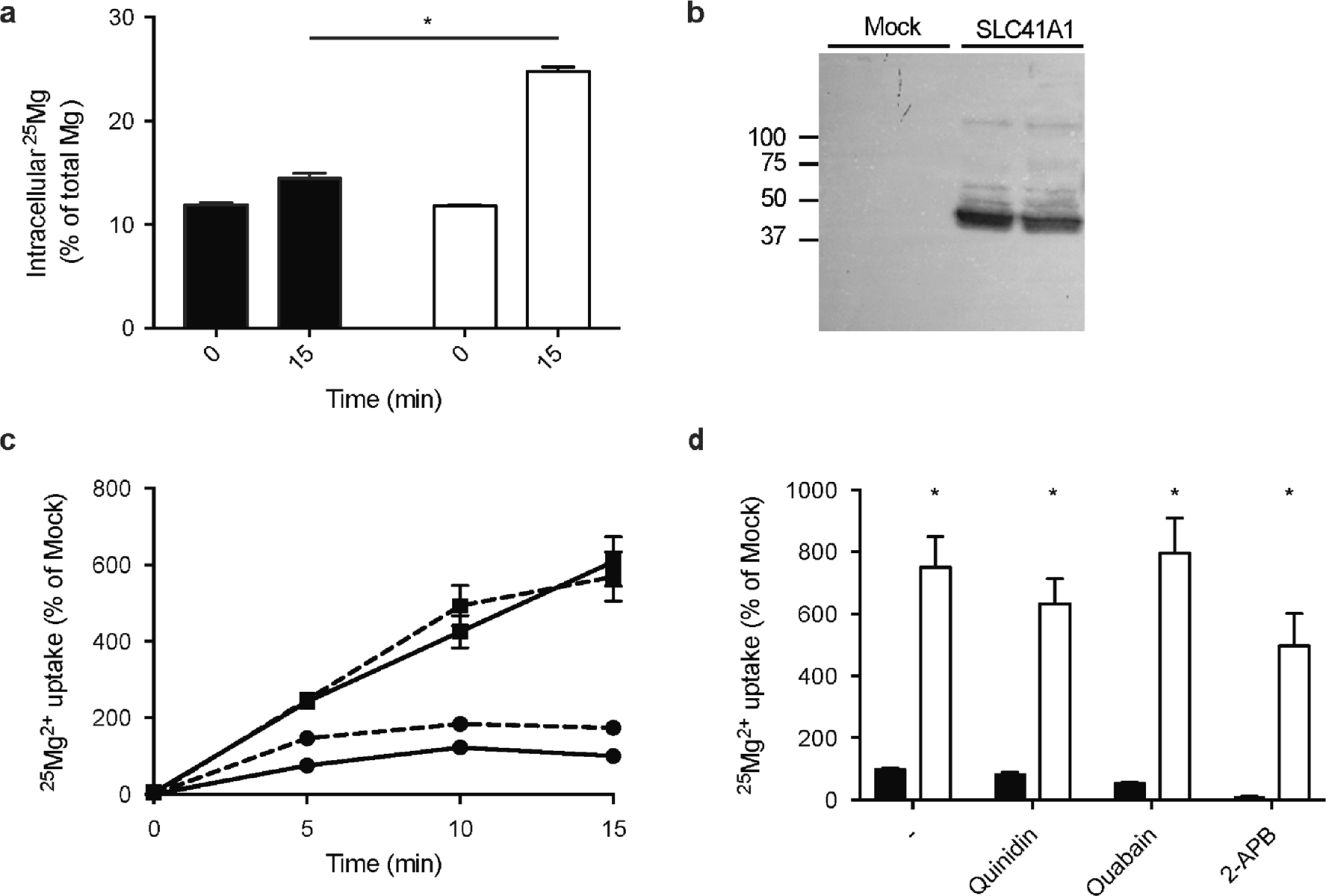
SLC41A1 mediates cellular Mg^2+^ uptake. **a** Intracellular ^25^Mg^2+^ as % percentage of total Mg^2+^ in mock (black bars) and SLC41A1 (white bars)-transfected HEK293 cells after 0 and 15 min of incubation in ^25^Mg^2+^. **b** Immunoblots showing expression of SLC41A1. The blots show a representative experiment of three independent experiments. **c** Time curve of ^25^Mg^2+^ uptake in mock and SLC41A1-transfected HEK293 cells in basic uptake buffer (solid line) and Na-gluconate containing uptake buffer (dashed line). All results are relative to the mock condition in the basic uptake buffer. **d**^25^Mg^2+^ uptake after 15 min in mock (black bars) and SLC41A1 (white bars)-transfected HEK293 cells in the presence of 100 μM quinidine, 100 μM ouabain, and 100 μM 2-APB. All results are relative to the mock condition in the basic uptake buffer. All graphs show the mean of three independent experiments ± SEM, *P < 0.05 compared to mock

Given that SLC41A1-mediated Mg^2+^ extrusion was not Na^+^-dependent in our setup, we examined whether SLC41A1 can facilitate Mg^2+^ uptake despite high extracellular Na^+^ concentrations using physiological conditions of 1 mM Mg^2+^ and 140 mM Na^+^ in the extracellular buffer. Indeed, SLC41A1 increases ^25^Mg^2+^ uptake fivefold compared to mock-transfected cells (Fig. 8a, b). Given that SLC41A1-mediated transport has been shown to be electroneutral and Na^+^ was excluded as a counter-ion, Cl^−^ was considered to be co-transported with Mg^2+^. However, SLC41A1-mediated ^25^Mg^2+^ uptake was unaffected by using a Na-gluconate buffer instead of NaCl (Fig. 8c). Moreover, SLC41A1 function could not be inhibited using 2-APB, an inhibitor of TRPM7 Mg^2+^ channels, ouabain, and quinidine, a non-selective Na^+^-channel blocker (Fig. 8d).

### SLC41A1 is expressed at the basolateral plasma membrane

**Fig. 9:**
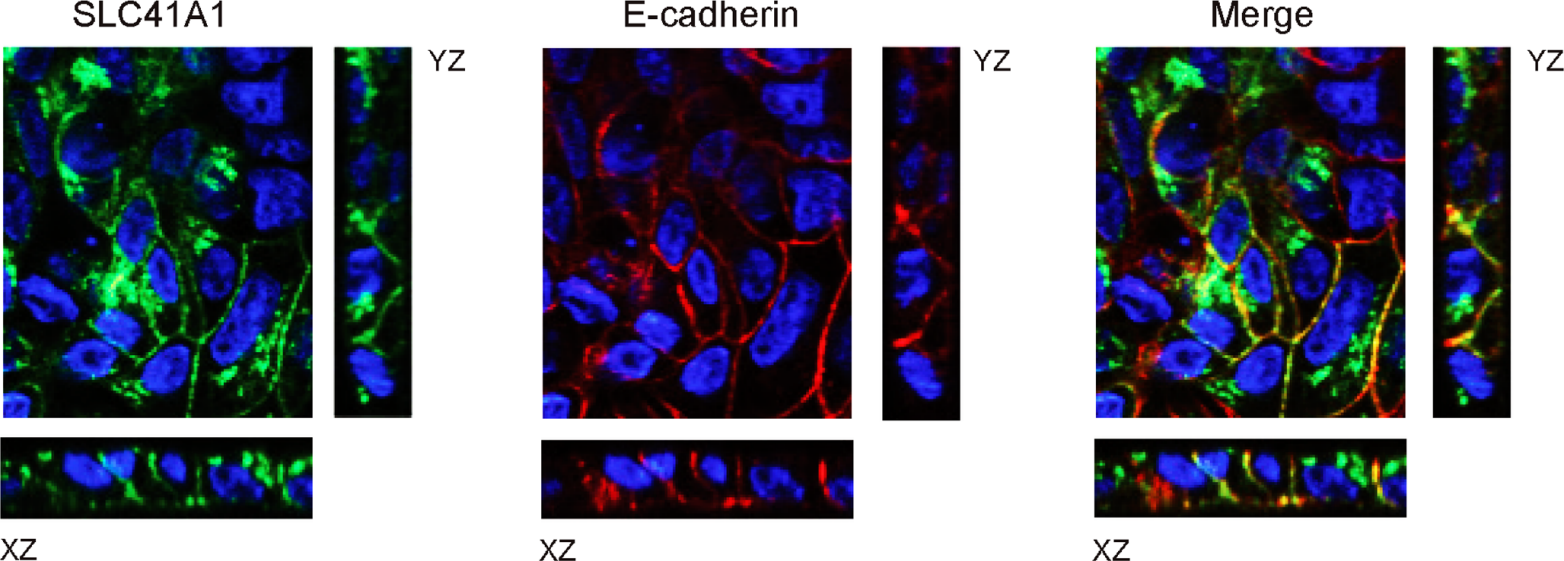
SLC41A1 is expressed at the basolateral membrane. Localization of SLC41A1 (green) and E-cadherin (red) in stably transfected polarized MDCKI cells with HA-tagged SLC41A1 is shown by immunofluorescence and confocal analyses in Z, XZ, and YZ sections. In blue, DAPI staining

SLC41A1 was previously shown to be expressed in TAL and DCT cells [17]. To examine its subcellular localization, MDCKI cells were stably transfected with HA-tagged SLC41A1. Immunocytochemical analysis demonstrated that HA-tagged SLC41A1 was expressed at the basolateral membrane, co-expressing with plasma membrane marker E-cadherin (Fig. 9). Additionally, HA-tagged SLC41A1 expression was detected in intracellular structures, possibly vesicles or the Golgi apparatus.

## Discussion

In the present study, we show that SLC41A1 is essential for Mg^2+^ homeostasis. Our main finding is that *slc41a1* knockdown causes a reduced Mg content in zebrafish larvae. Importantly, this Mg^2+^ deficiency can be rescued by expression of mammalian SLC41A1. Our results demonstrate that SLC41A1 is integral to the basolateral membrane and is involved in cellular Mg^2+^ uptake as well as extrusion in cultured renal epithelial cells. Based on our findings, we propose that SLC41A1 contributes to basolateral Mg^2+^ transport in the kidney.

The low Mg content in zebrafish *slc41a1* morphants points to a specific renal Mg^2+^ leak. The decrease in total Mg content can only be explained by renal (pronephric) Mg^2+^ wasting since the pronephros is the only exiting route for Mg^2+^ in developing zebrafish (0–120 hpf). In this time frame, there is no intestinal Mg^2+^ excretion since zebrafish do not eat and also do not drink as they are hyperosmotic with respect to the surrounding environment. In addition, by growing the zebrafish in a Mg^2+^-free E3 medium, skin Mg^2+^ uptake is abolished, so the lower Mg content in *slc41a1* morphants compared to controls can only be explained by deficient tubular Mg^2+^ reabsorption. In agreement with a defect in renal Mg^2+^ reabsorption, the total Mg content in *slc41a1* morphants was decreased after full maturation of the pronephros (96 and 120 hpf) but not at earlier time points [31].

Our cell experiments support a basolateral expression pattern of SLC41A1 that is in line with a Mg^2+^ extrusion function. As SLC41A1 is expressed in the DCT [17], where transcellular transport of Mg^2+^ occurs, it can be reasoned that SLC41A1 may contribute to the extrusion of Mg^2+^ in this segment of the nephron. In line with these findings, mRNA expression of genes involved in DCT Mg^2+^ reabsorption was upregulated in *slc41a1* morphants (*trpm6*, *slc12a3*, *cnnm2a*, *cnnm2b*, and *slc41a3* [9, 10]), further supporting a Mg^2+^ transport defect in the DCT. However, a function of SLC41A1 in the TAL cannot be discarded.

The renal Mg^2+^ leak in *slc41a1* morphants could not be explained by glomerular dysfunction and/or disturbances in renal fluid flow, because enlarged pericardial cavities (fluid retention) and pronephric cysts were absent in the fish where the total Mg content was measured. These kidney defects have been, however, associated with *slc41a1* knockdown in a study by Hurd et al. [17]. Differences in the doses of morpholino administered to the zebrafish embryos may explain this additional phenotype observed by Hurd et al. in *slc41a1* morphants.

The renal Mg^2+^ wasting phenotype in *slc41a1*-knockdown zebrafish was rescued by expression of mouse WT SLC41A1 but not by expression of SLC41A1-p.Asp262Ala, harboring a mutation in a key residue for SLC41A1 function [21]. This important finding proved the specificity of our loss-of-function approach. But more importantly, it indicated that the Mg^2+^ transport function of SLC41A1 is required to rescue the phenotype and that zebrafish Slc41a1 is functional equivalent to mammalian SLC41A1.

In line with SLC41A1 function in vivo, we demonstrate that SLC41A1 is a Mg^2+^ transporter that facilitates cellular Mg^2+^ uptake as well as Mg^2+^ extrusion dependent on the concentration gradient. In contrast to previous studies suggesting that SLC41A1 functions as a Na^+^-Mg^2+^ exchanger, our results do not support a Na^+^-dependent mechanism [17, 20]. Our findings show that SLC41A1 transports Mg^2+^ in Na^+^-free (NMDG-containing) buffer which cannot be blocked by Na^+^-channel blocker quinidine and Na^+^-K^+^-ATPase inhibitor ouabain.

The molecular mechanism of SLC41A1-mediated Mg^2+^ transport remains elusive. SLC41A1 function is not altered by ouabain, in line with an electroneutral transport mode and a Na^+^-independent mechanism. Our experiments exclude Cl^−^ as co-transported ion, but we were not able to identify the counter/co-ion. Based on the strong capacity of SLC41A1 to mediate both Mg^2+^ uptake and extrusion, the Mg^2+^ gradient seems to be the main determinant of Mg^2+^ transport. Previous studies have suggested the presence of a Mg^2+^-Mg^2+^ exchange mechanism in erythrocytes, where Mg^2+^ can compete with Na^+^ extracellularly for common binding sites in the erythrocyte Na^+^-Mg^2+^ exchanger [11, 14]. Although such a mechanism would only account for minimal Mg^2+^ exchange and its physiological relevance is questionable, we cannot exclude this possibility based on our data. It is interesting to note that the fivefold increase in Mg^2+^ uptake by SLC41A1 is high compared to previous experiments measuring CNNM2-dependent Mg^2+^ uptake (2–3-fold). In contrast to CNNM2-dependent Mg^2+^ uptake, the effects cannot be blocked by 2-APB, supporting that SLC41A1 mediates Mg^2+^ uptake and is not dependent on TRPM7.

An exon-skipping *SLC41A1* mutation has been previously associated with a nephronophthisis-like phenotype [17]. This study by Hurd et al. described two patients with a homozygous *SLC41A1* mutation that developed renal failure, eventually requiring renal transplantation [17]. These patients did not manifest hypomagnesemia and/or hypermagnesuria, symptoms expected upon a tubular Mg^2+^ transport impairment. However, it must be indicated that kidney failure typically results in Mg^2+^ conservation [7], an effect that may mask renal Mg^2+^ reabsorption defects in these specific *SLC41A1* patients. In contrast to the patient’s phenotype, no signs of renal cysts or dilatations were observed in our zebrafish model though our experiments were restricted to developing zebrafish (0–120 hpf). Furthermore, Mg^2+^ supplementation in the medium where zebrafish morphants were grown did not restore the Mg content to comparable levels as in controls. Similarly, in patients with inherited Mg^2+^ reabsorption disturbances and without renal cysts, Mg^2+^ homeostasis is not restored upon Mg^2+^ supplementation [2].

A strength of our study is that the reduced Mg content was present when inducing the *slc41a1* knockdown with both splice-site blocking and translation blocking MOs, a feature that was rescued by expression of mammalian SLC41A1. These results clearly demonstrate the specificity of the *slc41a1* knockdown in zebrafish to yield Mg^2+^ deficiency and discard these results as off-target effects of the *slc41a1* MO methodology used in the present study. A limitation is the use of HEK293 cells for our functional assays. However, Mg^2+^-transporting DCT cell lines are not available and our approach is more physiological than previous experiments. All measurements were performed in physiological Mg^2+^ concentrations (0.5–1 mM). Previous experiments were dependent on fluorescent probes and required super-physiological Mg^2+^ concentrations to load the cells (10 mM) [17, 20]. The use of stable isotopes is a more direct transport measurement than the use of fluorescent probes, which are sensitive to exchange with intracellular Mg^2+^ stores. An additional limitation of our study is that we cannot exclude differences in buffer capacity of the cells induced by overexpression of SLC41A1.

In conclusion, our data indicate that SLC41A1 regulates the body Mg^2+^ balance by transporting Mg^2+^ in the kidney. Further characterization of SLC41A1 in renal cells is required to determine the molecular mechanism of SLC41A1-mediated Mg^2+^ transport. Based on the SLC41A1 function disclosed, patients with hereditary hypomagnesemia should be screened for *SLC41A1* mutations.

## Electronic supplementary material

ESM 1 (PDF 629 kb)
