# Supplementary material for: SLC41A1 is essential for magnesium homeostasis in vivo
Source: Pflugers Arch. 2018 Nov 12;471(6):845–60. doi: 10.1007/s00424-018-2234-9 (PMC6533229; doi:10.1007/s00424-018-2234-9)
Supplement: Supplementary file 1 — (PDF 629 kb) [file 424_2018_2234_MOESM1_ESM.pdf]

**SUPPLEMENTARY INFORMATION**

# *SLC41A1* is essential for magnesium homeostasis *in vivo*

Francisco J. Arjona[1], Femke Latta[1], Sami G. Mohammed[1], Michael Thomassen[1], Erwin van Wijk[2], René J.M. Bindels[1], Joost G.J. Hoenderop[1,*], Jeroen H.F. de Baaij[1,*,#]

[1]Department of Physiology, Radboud Institute for Molecular Life Sciences, Radboud university medical center, Nijmegen, The Netherlands
[2]Department of Otorhinolaryngology, Donders Institute for Brain, Cognition and Behaviour, Radboud university medical center, Nijmegen, The Netherlands

[*] These authors contributed equally to this work.

[#] Corresponding author: Jeroen H.F. de Baaij
Telephone number: +31243614020
Email: Jeroen.deBaaij@radboudumc.nl

ORCID Francisco J. Arjona: 0000-0002-2479-0735
ORCID René J.M. Bindels: 0000-0003-1167-1339
ORCID Jeroen H.F. de Baaij: 0000-0003-2372-8486

```
Human SLC41A1         1 -------------MSSKPE---------PKDVHQLNG-TGPSASPCSSD----GPGREPL
Mouse SLC41A1         1 -------------MSSKPE---------PKDIHQPNG-TGPTPSPCSSD----GPGREPL
Rat SLC41A1           1 -------------MSSKPE---------PKDIHQPNG-TGPTPSPCSSD----GPGREPL
Zebrafish Slc41a1     1 MVLWLKDQDKLSDMSTGTERIEMKKEGVPPAYHHSNGSVHPVILPDSPEEVPQTPGEYEL

Human SLC41A1        34 AGTS---EFLGPDGAGVE-VVIESRANAKGVREEDALLENGSQSNESDDVSTDRGPAPPS
Mouse SLC41A1        34 AGTS---EFLGPDGVEV--VVIESRANAKGIREEDALLENGSQSNESDDVSTDRGPAPPS
Rat SLC41A1          34 AGTS---EFLGPDGAGVEVVVIESRANAKGVREEDALLENGSQSNESDDVSTDRVPAPPS
Zebrafish Slc41a1    61 TEVTSLPDCGDQENERPDMVVLDCRANAKGQREEDALLENASQSNESDDTSTDQSPVPPA

                                       TMD1
Human SLC41A1        90 PLKETSFSIGLQVLFPFLLAGFGTVAAGMVLDIVQHWEVFQKVTEVFILVPALLGLKGNL
Mouse SLC41A1        89 PLKETSFSIGLQVLFPFLLAGFGTVAAGMVLDIVQHWEVFQKVTEVFILVPALLGLKGNL
Rat SLC41A1          91 PLKETSFSIGLQVLFPFLLAGFGTVAAGMVLDIVQHWEVFQKVTEVFILVPALLGLKGNL
Zebrafish Slc41a1   121 PLKETSFSIGLQVLIPFLLAGFGTVAAGMVLDIVQHWTVFTEVTEVFILVPALLGLKGNL
                                                                       PX6GN
                                                              TMD2
Human SLC41A1       150 EMTLASRLSTAANIGHMDTPKELWRMITGNMALIQVQATVVGFLASIAAVVFGWIPDGHF
Mouse SLC41A1       149 EMTLASRLSTAANIGQMDTPKELWRMITGNMALIQVQATVVGFLASIAAVVFGWIPDGHF
Rat SLC41A1         151 EMTLASRLSTAANIGQMDTPKELWRMITGNMALIQVQATVVGFLASIAAVVFGWIPDGHF
Zebrafish Slc41a1   181 EMTLASRLSTAANIGQMDTAKDMWKMIMGNLALIQVQATVVGFLASIAAVIFGWIPEGNF

                                       TMD3                                TMD4
Human SLC41A1       210 SIPHAFLLCASSVATAFIASLVLGMIMIGVIIGSRKIGINPDNVATPIAASLGDLITLAL
Mouse SLC41A1       209 SIPHAFLLCASSVATAFIASLVLGMIMIGVIIGSRKIGINPDNVATPIAASLGDLITLAL
Rat SLC41A1         211 SIPHAFLLCASSVATAFIASLVLGMIMIGVIIGSRKIGINPDNVATPIAASLGDLITLAL
Zebrafish Slc41a1   241 RMGHAILLCASSVATAFIASLALGLIMIGVIIASRKVGINPDNVATPIAASLGDLITLSL
                                                                P(D/A)X4PX6D ↑
                                           TMD5                            TMD6
Human SLC41A1       270 LSGISWGLYLELNHWRYIYPLVCAFFVALLPVWVVLARRSPATREVLYSGWEPVIIAMAI
Mouse SLC41A1       269 LSGISWGLYLELKHWRYIYPLVCAFFVALLPVWVVLARRSPATREVLYSGWEPVIIAMAI
Rat SLC41A1         271 LSGISWGLYLELTHWRYIYPLVCAFFVALLPVWVVLARRSPATREVLYSGWEPVIIAMAI
Zebrafish Slc41a1   301 LAGISTGLYKELEFNNYANPMVCAFFVALTPIWVLIARRIPSTREVLYSGWEPVIIAMAI

                                            TMD7
Human SLC41A1       330 SSVGGLILDKTVSDPNFAGMAVFTPVINGVGGNLVAVQASRISTFLHMNGMPGENSEQAP
Mouse SLC41A1       329 SSVGGLILDKTVSDPNFAGMAVFTPVINGVGGNLVAVQASRISTFLHMNGMPGENSEPTP
Rat SLC41A1         331 SSVGGLILDKTVSDPNFAGMAVFTPVINGVGGNLVAVQASRISTFLHMNGMPGENSEPTP
Zebrafish Slc41a1   361 SSVGGLILDKTVSNPNFAGMAVFTPVINGVGGNLVAVQASRISTYLHMNALPIVEPNPAP

                                                    TMD8                   TMD9
Human SLC41A1       390 RRCPSPCTTFFSPDVNSRSARVLFLLVVPGHLVFLYTISCMQGGHTTLTLIFIIFYMTAA
Mouse SLC41A1       389 RRCPSPCTTFFSPDVNSRSARVLFLLVVPGHLVFLYTISCMQGGHTTLTLIFIIFYMTAA
Rat SLC41A1         391 RRCPSPCTTFFSPDVNSRSARVLFLLVVPGHLVFLYTISCMQGGHTTLTLIFIIFYMTAA
Zebrafish Slc41a1   421 RKCPTPWGTFFGSGVNSRSAKVLFLLVAPGHLVFLYTINSMRGGHTTLTYIFIAFYLAAA

                                                                  TMD10
Human SLC41A1       450 LLQVLILLYIADWMVHWMWGRGLDPDNFSIPYLTALGDLLGTGLLALSFHVLWLIGDRDT
Mouse SLC41A1       449 LLQVLILLYIADWMVHWMWGRGLDPDNFSIPYLTALGDLLGTGLLALSFHVLWLIGDRDT
Rat SLC41A1         451 LLQVLILLYIADWMVNWMWGRGLDPDNFSIPYLTALGDLLGTGLLALSFHVLWLIGDRDT
Zebrafish Slc41a1   481 LLQVIILLYLADWMVNWMWRRGMDPDNFSIPYLTALGDLLGTGFLALCFHILWLIGDRDT

Human SLC41A1       510 DVGD
Mouse SLC41A1       509 DVGD
Rat SLC41A1         511 DVGD
Zebrafish Slc41a1   541 DVGD
```

**Online Resource 1. Conservation of SLC41A1 proteins between zebrafish and mammals.** Alignment of the zebrafish Slc41a1 protein (GenBank accession no. XP_002663867) and its mammalian counterparts: human SLC41A1 protein (GenBank accession no. NP_776253), mouse SLC41A1 protein (GenBank accession no. NP_776290), and rat SLC41A1 protein (GenBank accession no. NP_001102325). Identical amino acids are

boxed in black, conservative substitutions in gray. The predicted transmembrane α-helices in the SLC41A1 proteins are indicated by TMD1-TMD10. The residues conforming the two functionally relevant motifs for the transport of $Mg^{2+}$, $PX_6GN$ and $P(D/A)X_4PX_6D$, are indicated. The residue mutated to generate a dysfunctional SLC41A1, and located in the pore region, is indicated by an arrow.
